# Evidence syntheses to support decision-making related to the Covid-19 pandemic

**DOI:** 10.11606/s1518-8787.2024058005226

**Published:** 2024-04-12

**Authors:** Keitty Regina Cordeiro de Andrade, Viviane Karoline da Silva Carvalho, Roberta Borges Silva, Cézar D. Luquine Junior, Cecília Menezes Farinasso, Cintia de Freitas Oliveira, Fabiana Mascarenhas, Gabriel Antônio Rezende de Paula, Isabela Porto de Toledo, Marina Arruda Melo Marinho, Virginia Kagure Wachira, Alessandra de Sá Earp Siqueira, Denizar Vianna Araújo, Camile Giaretta Sachetti, Daniela Fortunato Rêgo

**Affiliations:** I Ministério da Saúde Secretaria de Ciência, Tecnologia, Inovação e Insumos Estratégicos em Saúde Departamento de Ciência e Tecnologia Brasília DF Brasil Ministério da Saúde. Secretaria de Ciência, Tecnologia, Inovação e Insumos Estratégicos em Saúde. Departamento de Ciência e Tecnologia. Brasília, DF, Brasil

**Keywords:** Coronavirus, Implementation Science, Health Communication, Information Dissemination, Public Health

## Abstract

The COVID-19 pandemic generated a large volume of scientific productions with different quality levels. The speed with which knowledge was produced and shared worldwide imposed on health management the challenge of seeking ways to identify the best available evidence to support its decisions. In response to this challenge, the Department of Science and Technology of the Brazilian Ministry of Health started offering a service to produce and provide scientific knowledge addressing priority public health issues in the pandemic scenario. Drug treatments, non-pharmacological measures, testing, reinfection and immunological response, immunization, pathophysiology, post-COVID syndrome and adverse events are among the topics covered. In this article, we discuss the strengths and lessons learned, as well as the challenges and perspectives that present a real example of how to offer the best scientific evidence in a timely manner in order to assist the decision-making process during a public health emergency.

## INTRODUCTION

The public health crisis caused by the new coronavirus (COVID-19) has increased interest in access to the best evidence concerning policies, practices, and personal decisions. To support timely decision making, evidence synthesis experts summarized available research in a short period of time. The challenge was even greater due to the huge amount of publications available on COVID-19 in preprint articles and the speed with which scientific studies were conducted in the period^1^.

Evidence syntheses are summaries with interpretations of individual research, which answer specific questions within a larger context of knowledge^2,3^ and are essential for providing the highest level of evidence. However, their elaboration period, in many cases, exceeds the time to inform decisions during public health crises^4^. In these circumstances, rapid evidence syntheses are recommended^5,6^.

There is a growing body of literature on the use of on-demand evidence rapid response services and the limitations of their methods compared to other types of syntheses^6^. In this article, we report the initiative of a Brazilian team to provide the best scientific evidence, via a rapid response service within a federal agency, the Ministry of Health (MS)^12,13^. During the first two years of the pandemic, the team conducted around 90 studies, covering 20 technical areas. The lessons learned and the challenges overcome during these studies are presented below.

### Facilitators in the Process of Responding to Demands for Evidence

**Figure 1:**
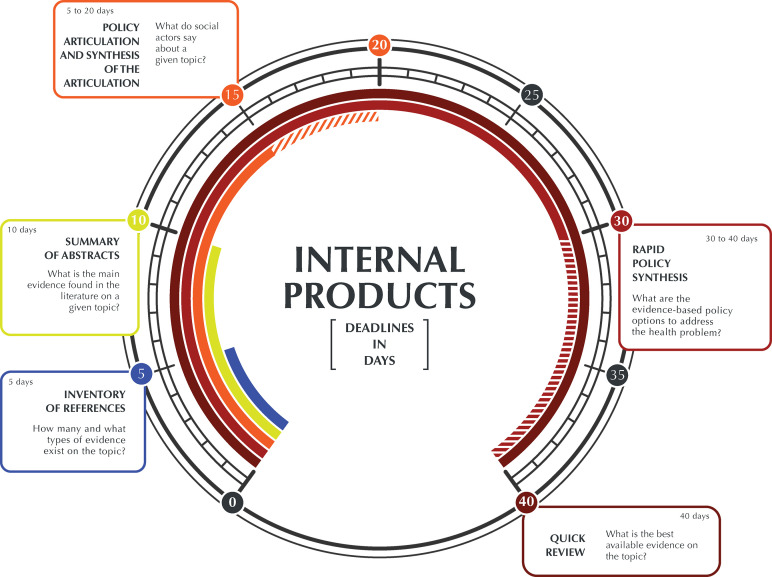
Portfolio with rapid response studies. Note: (a) Reference inventory: identifies and quantifies available evidence on a given topic; (b) Summary of abstracts: categorizes, quantifies and summarizes the results contained in the selected abstracts on a given topic; (c) Rapid review: expedites the process of conducting a traditional systematic review by simplifying or omitting specific methods to produce evidence for stakeholders in a resource-efficient manner; (d) Rapid synthesis for policies: presents a synthesis of evidence on policy options and considerations on their implementation and equity analysis; (e) Policy dialogue and dialogue synthesis: collects colloquial evidence from social actors involved in the issue and synthesizes the evidence collected in the dialogue.

There was an experienced and multidisciplinary team dedicated to work on evidence syntheses. This team created a portfolio that functioned as a service charter, presenting the types of evidence rapid responses products that could be developed, their objectives, limitations, elaboration deadlines, and the necessary request flows^14^ (Figure 1). The team also knew how many members were needed to complete a synthesis within a specific deadline, as well as to determine the choice of the type of synthesis to be prepared, the type of study design to be included and the evidence sources^14^.

The continuous relationship between those preparing the syntheses and the decision makers was important to generate trust and help in refining the main doubts regarding the fight against the pandemic, and also in the definition of the eligibility criteria and results of interest for research. Research demands were co-created by the team and consumers, which strengthened the service, characterizing it as a collaborative knowledge translation^15^. These decision makers, in general, were from final technical areas of the Ministry of Health and carried out the action in different ways, involving primary and specialized health care and mainly health surveillance—epidemiological, laboratory and worker health.

With the aim of aligning expectations and optimizing time, one established a workflow, which consisted of sequential steps: receiving demand via the Electronic Information System (SEI) and through direct contact with the area coordination; alignment meeting between the team and decision makers; study development; preparation of the communication product, and demand delivery. Each of them had its own flows and schedules adapted to the demanding areas’ needs.

Frequently, the demanding areas’ time to align research questions was reduced, but efforts were directed at clarifying the question, target audience and the usefulness of research results. Furthermore, this strategy contributed to increasing the engagement of decision makers in some steps of synthesis preparation, that is, it was a co-creation process, which could facilitate the evidence absorption in decision making.

Requests were prioritized based on: response urgent need; level of decision-making, often measured by the hierarchical position of the demanding area; team’s knowledge and technical capacity regarding the content, and evidence availability, verified by a preliminary database search. The team was also proactive in searching for immediate and long-term priorities, anticipating what might be needed.

The types of evidence products developed varied in relation to the scope (public health measures, clinical management, health systems arrangements, and economic and social responses), time required for response and availability of the team, which made some adjustments and methodological shortcuts to deliver products faster without scientific rigor reduction (Table 1)^5^. As science evolved quickly in the pandemic scenario, some syntheses were periodically updated. Changes were presented to the decision maker in a quick and transparent manner, through a text box with highlights entitled “Main updates to this version” at the beginning of the synthesis. The main evidence products included rapid systematic reviews (34%), abstract summaries (25%), and reference inventories (23%) (Table 2) ^7,14^.

**Table 1:** Specific steps to conducting rapid reviews.

Steps
Question definition
Limit the number of results, focusing on those most important for decision making
Protocol elaboration
Develop a brief protocol to reduce duplication and, if possible, provide a publicly available record prior to carrying out the review
Literature search
Narrow the search strategy (database numbers, gray literature, search terms, date, setting, and language, for example)
Limit the types of studies included to those that will provide the most rigorous evidence to answer the question (such as only systematic reviews, and if none exist, search for other designs such as randomized controlled trials, etc.)
Consult an information specialist/librarian
Study selection
Use a single reviewer experienced in preparing systematic reviews, with or without validation by a second reviewer
Incorporate the use of software (as Covidence, DistillerSR, EPPI-Reviewer, Crowdsourcing, Rayyan) to expedite the process
Data extraction
It may be by a single reviewer experienced in preparing systematic reviews, with or without validation by a second reviewer
Consider using data from existing systematic reviews
Use data extraction tools (with or without data mining)
Methodological quality and risk of bias evaluation
It may be by a single reviewer experienced in preparing systematic reviews, with or without validation by a second reviewer
Choose the appropriate assessment instrument for each type of study
Use the Grading of Recommendations Assessment, Development and Evaluation (GRADE) or Confidence in the Evidence from Reviews of Qualitative Research (GRADE CERQual) system; it may be also by a single reviewer
Evidence synthesis
Descriptive synthesis is the most common method; quantitative synthesis (as meta-analysis) will depend on available time and resources
Provide conclusions, implications for decision-making (for practice), implications for future research, and discuss potential review limitations
Dissemination
Package knowledge depending on the specific target audience (politicians, healthcare professionals, civil society, for example)
Summarizing evidence in a short format (such as one page) can make results easier to absorb with key messages highlighted for the decision-making process
Use visual resources like infographics
Consider targeted dissemination means such as podcasts, YouTube, LinkedIn, Twitter, ResearchGate and media releases
Update
Update the synthesis periodically
Present at the beginning of the updated synthesis “Main updates to this version” (date and search strategy, results, for example)

Source: Adapted from World Health Organization (WHO)^5.^

**Table 2:** Evidence syntheses carried out during the COVID-19 pandemic by type of study.

Titles of syntheses carried out	n (%)
Previous literature search	2 (2)
Scientific evidence on the effectiveness and safety of booster doses of COVID-19 vaccines	
New monoclonal antibody therapy for treating COVID-19	
Reference inventory	21 (23)
International recommendations for necropsy in cases of deaths from COVID-19	
Association between the use of angiotensin-converting enzyme inhibitors (ACEI), angiotensin-converting enzyme 2 (ACE2), and Ibuprofen and COVID-19 patients’ safety	
Effects of social distancing measures during the COVID-19 pandemic	
Pharmacological therapeutic alternatives for mild COVID-19 cases	
Effectiveness of using unmanned aerial vehicles in disinfecting public places	
Effect of pharmacological prophylactic treatment for COVID-19	
Effect of pharmacological prophylactic treatment for COVID-19: update	
Effects of pharmacological treatment for the initial phase of COVID-19 (up to five days from the onset of symptoms)	
Effects of chloroquine and hydroxychloroquine for pre-hospital and/or outpatient treatment of COVID-19 patients	
Effects of ivermectin for pre-hospital or outpatient treatment of COVID -19 patients	
Effects of nitazoxanide for pre-COVID-19 treatment	
Natural history of COVID-19	
Efficacy, effectiveness, and safety of pharmacological treatment of mild and moderate COVID-19 patients	
Systematic reviews on worsening factors and mortality from COVID-19	
Health safety at sporting events in the context of the COVID-19 pandemic	
Assessment of the efficacy and safety of COVID-19 vaccination in pregnant and postpartum women	
Efficacy and safety of the fourth dose of the COVID-19 vaccine: 1^st^ update	
Efficacy and safety of the fourth dose of the COVID-19 vaccine: 2^nd^ update	
Occurrence of myocarditis in children after COVID-19 vaccination	
Prioritized research topics for COVID-19 to support the launch of call for proposals for the vaccine	
Efficacy and safety of the Coronavac vaccine	
Technical note	10 (11)
Chloroquine or hydroxychloroquine (associated or not with azithromycin) for the early treatment of Covid-19 patients and its 13 updates	
Occurrence of post-COVID-19 clinical manifestations	
International evidence and recommendations regarding COVID-19 vaccination in children aged between 5 and 11 years	
Occurrence of post-COVID-19 clinical manifestations: update	
Use of the prothrombin activity time test in COVID-19	
Effects of the ciclesonide as an aid in coping with COVID-19	
Effects of gargling with iodine on COVID-19 and early viral clearance among COVID-19 patients when gargling with povidone-iodine and essential oils	
Technical-scientific dossier on the use of chlorine dioxide for COVID-19	
Feasibility study of adopting a comprehensive outpatient treatment protocol against COVID-19	
Use of corynebacterium parvum in the context of COVID-19	
Rapid systematic review	31 (34)
Therapeutic alternatives for treating human coronavirus	
Therapeutic alternatives for treating human coronavirus: 1^st^ update	
Therapeutic alternatives for treating human coronavirus: 2^nd^ update	
Efficacy and safety of chloroquine and hydroxychloroquine (alone or in combination) for treating human coronavirus infections	
Efficacy and safety of chloroquine and hydroxychloroquine (alone or in combination) for treating human coronavirus infections: 1^st^ update	
Efficacy and safety of chloroquine and hydroxychloroquine (alone or in combination) for treating human coronavirus infections: 2^nd^ update	
Efficacy and safety of chloroquine and hydroxychloroquine (alone or in combination) for treating human coronavirus infections: 3^rd^ update	
Efficacy and safety of chloroquine and hydroxychloroquine (alone or in combination) for treating human coronavirus infections: 4^th^ update	
Efficacy and safety of chloroquine and hydroxychloroquine (alone or in combination) for treating human coronavirus infections: 5^th^ update	
Effectiveness of social distancing in epidemics	
Efficacy and safety of convalescent plasma for treating SARS-CoV-2 infections	
Efficacy of disinfection of N95, FFP2 and FFP3 face masks/respirators for safe reuse in the prevention of respiratory infections	
Efficacy and safety of convalescent plasma for treating SARS-CoV-2 infections: 1^st^ update	
Viral activity of human coronavirus on household and hospital surfaces	
Efficacy and safety of convalescent plasma for treating SARS-CoV-2 infections: 2^nd^ update	
Sanitary and non-sanitary effects of social distancing measures during the COVID-19 pandemic	
Efficacy and safety of convalescent plasma for treating SARS-CoV-2 infections: 3^rd^ update	
Effects of pharmacological treatment for the initial phase of COVID-19 (up to five days from the onset of symptoms)	
Effect of pharmacological prophylactic treatment for COVID-19	
Efficacy and safety of convalescent plasma for treating SARS-CoV-2 infections: 4^th^ update	
Rapid systematic review on the effects of chloroquine and hydroxychloroquine for pre-hospital and/or outpatient treatment of COVID-19 patients	
Rapid systematic review on cases of SARS-CoV-2 reinfection	
Rapid systematic review on immunological response to SARS-CoV-2	
Rapid systematic review on cases of SARS-CoV-2 reinfection: 1^st^ update	
Efficacy, effectiveness, and safety of pharmacological treatment of mild and moderate COVID-19 patients	
Identification of risk factors for worsening and mortality from COVID-19	
Assessment of the efficacy and safety of COVID-19 vaccination in pregnant and postpartum women	
Vaccination safety in adolescents	
Effectiveness and durability of the immune response against SARS-CoV-2	
Serial testing for COVID-19 in a school environment	
Efficacy and safety of the Coronavac vaccine in children	
Traditional systematic review	1 (1)
Efficacy/effectiveness of using medical and non-medical face masks, in open or closed environments, for those immunized (vaccinated and/or convalescent) for COVID-19	
Rapid Synthesis for policies	1 (1)
Rapid synthesis for policies on strategies for gradual return from social distancing	
Summary of abstracts	22 (25)
Vitamin D and SARS-CoV-2 infections	
Temperature threshold for screening in convivial environments	
Recommended minimum physical distance in social environments	
Mapping of definitions of social distancing in the context of the COVID-19 pandemic	
Mapping of COVID-19 case definitions	
Guidelines for isolation in the context of COVID-19	
Mapping of terms to define severity of COVID-19 cases	
Target period for testing asymptomatic contacts of confirmed COVID-19 cases	
Mapping of evidence on pathophysiological changes in the air transportation of COVID-19 patients and other respiratory diseases	
Mapping of evidence on national and international recommendations for COVID-19 vaccination in pregnant women, postpartum women, breastfeeding women, and children	
Effectiveness of using face masks to prevent COVID-19	
Effects of Covid-19 vaccination in pregnant women, postpartum women, breastfeeding women, and children	
Mapping of evidence on national and international recommendations for COVID-19 vaccination in pregnant women, postpartum women, breastfeeding women and children	
Identification of the ideal testing interval to differentiate COVID-19 flu syndrome events	
Mapping of definitions of post-COVID-19 syndrome	
Effectiveness of the COVID-19 vaccination campaign to reduce bed occupancy in ICU	
Efficacy and safety of COVID-19 vaccination in adolescents	
Efficacy and safety of COVID-19 vaccination in adolescents: 1^st^ update	
National and international strategies for certification of COVID-19 immunization	
Effectiveness of the COVID-19 vaccination campaign in reducing bed occupancy in ICU	
To identify and summarize studies that evaluate the comparative effectiveness and safety of treatments for COVID-19	
Complementation regarding effective technical actions in the study of vitamin D to combat COVID-19	
Non-pharmacological alternatives to COVID-19	

ICU: intensive care unit.

**Figure 2:**
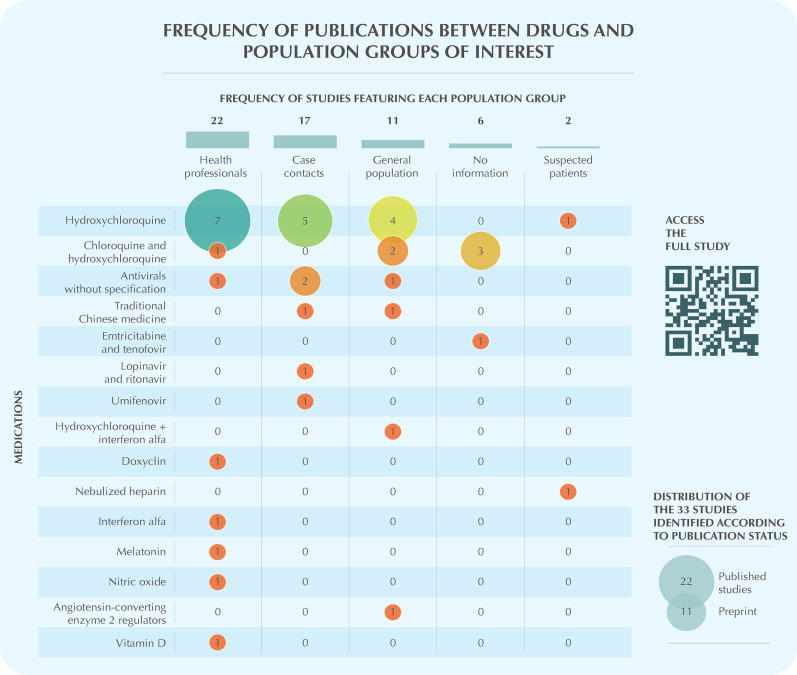
Example of an infographic generated from the results of a reference inventory. Note: The reference inventory aimed to identify and categorize published articles and ongoing studies on the effects of pharmacological prophylaxis for COVID-19. This study does not perform an analysis of main results, nor does it evaluate the methodological quality of the articles included. Furthermore, it does not represent an official recommendation from the Ministry of Health on the topic under consideration.

To make it usable, better packaging of knowledge was necessary. Besides the traditional format, an additional version was prepared by a scientific communication and information design team, responsible for making the language and format appropriate and accessible to the target audience. Scientific jargon was transformed into plain language, besides investment in visual summaries, such as infographics (Figure 2).

A knowledge intermediary was constantly used to transfer the evidence generated, in order to promote greater absorption of knowledge by potential users^16^.

It was a neutral communicator—like a necessary channel—between the generator and the potential knowledge user. People operating as intermediaries can pave the way for the absorption and potential implementation of scientific knowledge in policy due to their credibility^20^.

An example of a situation in which the evidence synthesis indicated that it was contrary to the use of a medicine and was not followed by some Brazilian managers was the case of hydroxychloroquine in the treatment for COVID-19^24^. In contrast, an example of implementation of the prepared syntheses was the summary of abstracts on mapping of definitions of post-COVID-19 syndrome, which contributed to the adoption of terminology related to post-COVID conditions and the definition of a new ICD (International Statistical Classification of Diseases and Health-Related Problems)^25^.

**Figure 3:**
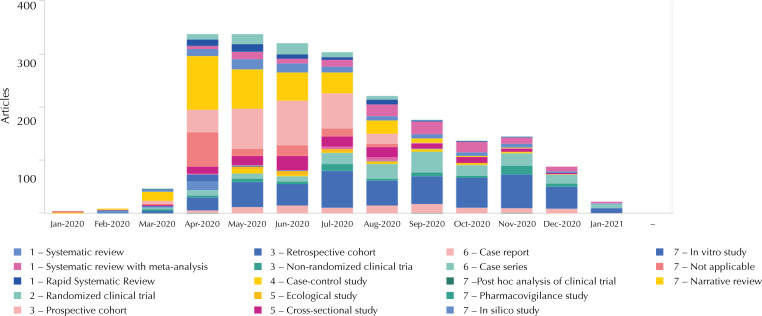
Dashboard of evidence on COVID-19 pharmacological treatment and vaccines.

A daily report of scientific evidence on pharmacological treatment for COVID-19 was established as a means of dissemination. The studies were screened, summarized, and classified by methodological design, as well as assessed for their methodological quality and risk of bias, using appropriate tools for each type of study, and, finally, made available in a dashboard (Figure 3). In this way, abstracts of preprint technical-scientific publications from indexed journals on the efficacy, safety and effectiveness of medicines and biological products used for COVID-19 treatment and prevention were made available daily. At the beginning of the pandemic, when there was a surge in publications and media coverage, the aim of this initiative was to promote access to evidence in a qualified, easy, and reliable manner.

There were sufficient organizational resources to respond to requests from decision makers. Part of the team used personal computers and Internet connections since they were working remotely; nevertheless, the department had the capacity for data storage and access to information sources upon payment of a license to use indexed journals publications.

The team sought to reduce the duplication of efforts in conducting syntheses by searching for ongoing research initiatives worldwide that addressed Brazilian decision makers’ doubts. For example, the group accessed the COVID-19 Evidence Network to support Decision-making (COVID-END) and the Brazilian Evidence-Informed Policy Network (Evipnet-Brasil). A prior protocol was also drawn up and, when possible, registered on public platforms.

### Challenges and Perspectives in Preparing Evidence Syntheses

Using evidence synthesis assists the decision-making process, but it is not sufficient to ensure evidence-informed decision making. Political and economic interests interfere in the process, and, therefore, the presence of institutional leadership that values the use of evidence facilitates its adoption^27^. Furthermore, uncertainty surrounded a scenario of incipient knowledge.

There was a kind of “evidence explosion” and many organizations began to promote themselves by producing documents based on it. There was no methodological consensus regarding the synthesis and evaluation of this evidence, and decision makers were not always able to differentiate a systematic review from a manual selection of studies.

It was common for several working groups in the country to develop the same themes. It is necessary to improve coordination and national communication tools, and mobilization must prioritize themes and disseminate results, avoiding duplication of efforts in preparing evidence syntheses.

The team was asked to deal with different sources of information, as there were policymakers interested in identifying evidence related to institutional documents from different countries to understand their experience in managing the pandemic.

Translation of evidence synthesis or documents prepared in other languages into Portuguese was a resource that was little used or took a long time. There was also a lack of an online platform to produce living documents and indicate which research efforts became obsolete.

Researchers used to scientific production understand how evidence evolves, so changes are seen as part of the process. However, for the lay public this process can be confusing and cause distrust. Thus, one issue faced was the lack of strategies to deal with studies that report results from the beginning of the pandemic, which could no longer be relevant, given the knowledge of the most recent COVID-19 situation. For example, a rapid review of social distancing in the transmission of the new coronavirus was maintained and studies continued to be published, reporting results from the beginning of the pandemic. Nevertheless, as much more was known about virus transmission, infection and prevention measures, these studies might not provide relevant value.

One of the department’s biggest challenges was engaging social actors interested in the topics of evidence syntheses, especially due to limited deadlines. Such involvement could help identify health needs and research problems, besides enabling assessments and finding interpretation. For example, they can provide their perspective on the issue, the results, the analysis interpretation, and the summary in plain language. Most are interested in various areas of research, but not everyone has experience in evidence synthesis. A useful strategy would be to provide those interested with adequate training on evidence-based health concepts and terms^28^.

It is noteworthy that given the resources available, the team always sought to make the most assertive decisions for the scenario, but other efforts also need to be encouraged. Besides contributions to the preparation of syntheses, it is necessary to inform the population about the results of the syntheses and recognize reliable scientific evidence. Initiatives in this area can contribute to the democratization of science and improvement of critical thinking about information published on the Internet. Furthermore, although evidence is fundamental to informing decisions, it is not the only factor considered in decision making. Nevertheless, one believes that the lack of alignment between the spheres of government may have compromised the implementation of the syntheses.

## FINAL CONSIDERATIONS

An important step towards the application of scientific knowledge in real time in Brazil was taken during the COVID-19 pandemic. It is insufficient to invest in selected research, and it is also necessary to encourage the use and creation of living online platforms. For this purpose, we must work in a network to avoid overlapping efforts and non-dissemination of the knowledge produced. More research is needed on how to present and prioritize results and how to engage the stakeholders in rapid syntheses to ensure all perspectives are achieved. Thus, the need to institutionalize the transparent and systematic use of evidence in decision-making processes stands out. This path taken by the group during the pandemic can be considered a proposal for an innovative model regarding the use of evidence syntheses to support health decision-making.
