# Supplementary material for: Evidence syntheses to support decision-making related to the Covid-19 pandemic
Source: Rev Saude Publica. 2024 Apr 12;58:16. doi: 10.11606/s1518-8787.2024058005226 (PMC11037906; doi:10.11606/s1518-8787.2024058005226)
Supplement: Translation

# Translation: Sínteses de evidências para apoio à tomada de decisões relacionadas à pandemia de covid-19

**Authors:** Keitty Regina Cordeiro de Andrade, Viviane Karoline da Silva Carvalho, Roberta Borges Silva, Cézar D. Luquine Junior, Cecília Menezes Farinasso, Cintia de Freitas Oliveira, Fabiana Mascarenhas, Gabriel Antônio Rezende de Paula, Isabela Porto de Toledo, Marina Arruda Melo Marinho, Virginia Kagure Wachira, Alessandra de Sá Earp Siqueira, Denizar Vianna Araújo, Camile Giaretta Sachetti, Daniela Fortunato Rêgo

**Affiliations:** I Brasil Ministério da Saúde. Secretaria de Ciência, Tecnologia, Inovação e Insumos Estratégicos em Saúde. Departamento de Ciência e Tecnologia. Brasília, DF, Brasil

**Keywords:** Coronavírus, Ciência da Implementação, Comunicação em Saúde, Disseminação de Informação, Saúde Pública

## Abstract

A pandemia de covid-19 gerou um numeroso volume de produções científicas com diferentes níveis de qualidade. A velocidade com que o conhecimento era produzido e compartilhado a nível mundial impôs à gestão em saúde o desafio de buscar meios de identificar as melhores evidências disponíveis para subsidiar suas decisões. Em resposta a este desafio, o Departamento de Ciência e Tecnologia do Ministério da Saúde do Brasil estabeleceu um serviço para elaborar e disponibilizar conhecimento científico abordando questões prioritárias de saúde pública no cenário da pandemia. Entre os temas abordados estão tratamentos medicamentosos, medidas não farmacológicas, testagem, reinfecção e resposta imunológica, imunização, fisiopatologia, síndrome pós-covid e eventos adversos. Neste artigo, discute-se os pontos fortes e lições aprendidas, bem como os desafios e perspectivas que fornecem um exemplo real sobre como disponibilizar as melhores evidências científicas, em tempo hábil e de forma oportuna, para auxiliar o processo decisório durante uma emergência em saúde pública.

## INTRODUÇÃO

A crise de saúde pública provocada pelo novo coronavírus (covid-19) aumentou o interesse no acesso às melhores evidências para informar políticas, práticas e decisões pessoais. Para apoiar a tomada de decisão oportuna, especialistas em sínteses de evidências resumiram a pesquisa disponível em prazos curtos. O desafio foi ainda maior com a explosão da disponibilidade de publicações sobre covid-19 em artigos em pré-impressão, e da celeridade com que estudos científicos ocorreram no período^1^.

Sínteses de evidências são resumos com interpretações de pesquisas individuais, que respondem a questões específicas dentro de um contexto maior de conhecimento^2,3^, e são essenciais por fornecerem o mais alto nível de evidência. Contudo, o seu período de produção, em muitos casos, excede o tempo para informar decisões durante crises de saúde pública^4^. Nessas circunstâncias, as sínteses rápidas de evidências são recomendadas^5,6^.

Há uma literatura em expansão sobre o uso de serviços de resposta rápida de evidências sob demanda e as limitações de seus métodos, em comparação com os outros tipos de sínteses^6^. Neste artigo, relata-se a iniciativa de uma equipe brasileira para disponibilizar as melhores evidências científicas, por meio de um serviço de resposta rápida dentro de um órgão federal, o Ministério da Saúde (MS)^12,13^. Durante os dois primeiros anos da pandemia, a equipe elaborou cerca de 90 estudos, atendendo a 20 áreas técnicas, assim, apresentam-se a seguir as lições aprendidas e os desafios superados durante a realização destes estudos.

### Facilitadores no Processo de Resposta às Demandas por Evidências

Havia uma equipe experiente e multiprofissional na elaboração de sínteses de evidências. Este grupo criou um portfólio que funcionou como uma carta de serviços, apresentando os tipos de produtos de evidências que poderiam ser realizadas, seus objetivos, limitações, prazos para elaboração e os fluxos necessários para solicitação^14^ (Figura 1). Sabia, também, quantos membros precisavam para concluir uma síntese dentro de um prazo específico, assim como para determinar a escolha do tipo de síntese a ser elaborada, o tipo de delineamento do estudo a ser incluído e as fontes de evidência^14^.

**Figura 1:** Portfólio com estudos de respostas rápidas. Nota: (a) Inventário de referências: identifica e quantifica evidências disponíveis sobre determinada temática; (b) Sumário de resumos: categoriza, quantifica e sumariza os resultados contidos nos resumos selecionados sobre determinada temática; (c) Revisão rápida: acelera o processo de condução de uma revisão sistemática tradicional por meio da simplificação ou omissão de métodos específicos para produzir evidências para as partes interessadas de maneira eficiente em termos de recursos; (d) Síntese rápida para políticas: apresenta uma síntese de evidências sobre opções políticas e de considerações sobre sua implementação e análise de equidade; (e) Diálogo de políticas e síntese do diálogo: realiza a coleta de evidências coloquiais dos atores sociais envolvidos na questão e sintetiza as evidências coletadas no diálogo.

O relacionamento contínuo entre os elaboradores das sínteses e os tomadores de decisão foi importante para gerar confiança e auxiliar no refinamento das principais dúvidas no enfrentamento da pandemia, bem como na definição dos critérios de elegibilidade e resultados de interesse para pesquisas. As demandas de pesquisa eram cocriadas entre os elaboradores e consumidores, o que fortaleceu o serviço, caracterizando-o como colaborativo de *knowledge translation*^15^*.* Esses tomadores de decisão, em geral, eram de áreas técnicas finalísticas do MS, que realizavam a ação de maneira diversa, envolvendo a atenção primária e especializada à saúde e principalmente a vigilância em saúde – epidemiológica, laboratorial e saúde do trabalhador.

Com o objetivo de alinhar as expectativas e otimizar o tempo, estabeleceu-se um fluxo de trabalho que consistia em etapas sequenciais: recebimento da demanda via Sistema Eletrônico de Informações (SEI) e por meio de contato direto com a coordenação da área; reunião de alinhamento entre os elaboradores e tomadores de decisão; produção do estudo; elaboração do produto de comunicação; e entrega da demanda. Cada uma delas contava com fluxos próprios e cronogramas adaptados à necessidade das áreas demandantes.

Com frequência, o tempo dos demandantes para alinhamentos das perguntas de pesquisa era reduzido, mas todo esforço era válido para se ter lucidez sobre a pergunta, o público de interesse e a utilidade dos resultados da pesquisa. Além disso, essa estratégia contribuiu para aumentar o engajamento dos tomadores de decisão em algumas etapas da elaboração das sínteses, ou seja, um processo de cocriação, o que poderia facilitar a absorção das evidências na tomada de decisão.

As solicitações eram priorizadas a partir da necessidade de urgência de resposta; do nível da tomada de decisão, muitas vezes medido pela posição hierárquica do demandante; do conhecimento e capacidade técnica da equipe sobre o conteúdo; e da disponibilidade das evidências, por meio de busca preliminar em base de dados. A equipe também foi proativa na busca por prioridades imediatas e a longo prazo, prevendo o que poderia vir a ser necessário.

Os tipos de produtos de evidências elaborados variavam em relação ao escopo (medidas de saúde pública, gestão clínica, arranjos de sistemas de saúde e respostas econômicas e sociais), ao tempo requerido para resposta e à disponibilidade da equipe, que realizava alguns ajustes e atalhos metodológicos para encurtar o tempo na entrega dos produtos sem reduzir o rigor científico (Tabela 1)^5^. Como a ciência evoluiu de maneira rápida no cenário da pandemia, realizava-se atualização periódica de algumas sínteses. Utilizava-se a estratégia de apresentação das mudanças para o tomador de decisão, de forma rápida e transparente, por meio de caixa de texto com destaques intitulados “Principais atualizações desta versão” no início da síntese. Os principais produtos de evidência incluíram revisões sistemáticas rápidas (34%), sumários de resumos (25%) e inventários de referências (23%) (Tabela 2)^7,14^.

**Tabela 1:** Etapas específicas para elaboração de revisões rápidas.

Etapas
Definição da pergunta
Limitar o número de resultados, com foco naqueles mais importantes para a tomada de decisão
Elaboração de protocolo
Elaborar breve protocolo para diminuir a duplicação e, se possível, fornecer o registro disponível publicamente previamente a realização da revisão
Busca na literatura
Restringir a estratégia de pesquisa (por exemplo, números de bases de dados, literatura cinzenta, termos de buscas, data, cenário e idioma)
Limitar os tipos de estudos incluídos que irão fornecer a evidência mais rigorosa para responder à pergunta (por exemplo, apenas revisões sistemáticas, caso não existam, pesquisar outros delineamentos como ensaio clínico randomizado etc.)
Contar com um especialista em informação/bibliotecário
Seleção dos estudos
Usar um único revisor experiente na elaboração de revisões sistemáticas, com ou sem validação por um segundo revisor
Incorporar o uso de softwares (por exemplo, Covidence, DistillerSR, EPPI-Reviewer, Crowdsourcing, Rayyan) para agilizar o processo
Extração dos dados
Pode ser realizada por único revisor, experiente na elaboração de revisões sistemáticas, com ou sem validação de um segundo revisor
Considerar o uso de dados de revisões sistemáticas existentes
Usar ferramentas de extração de dados (com ou sem recursos de mineração de dados)
Avaliação da qualidade metodológica e risco de viés
Pode ser realizada por único revisor, experiente na elaboração de revisões sistemáticas, com ou sem validação de um segundo revisor
Eleger o instrumento de avaliação adequada para cada tipo de estudo
Usar o sistema *Grading of Recommendations Assessment, Development and Evaluation* (GRADE) ou *Confidence in the Evidence from Reviews of Qualitative Research* (GRADE CERQual) também por um único revisor
Síntese das evidências
Sintetizar descritivamente é o método mais comum; síntese quantitativa (por exemplo, metanálise) dependerá de tempo e recursos disponíveis
Fornecer conclusões, implicações para o processo decisório (para a prática), implicações para futuras pesquisas e discutir potenciais limitações da revisão
Disseminação
Empacotar o conhecimento a depender do público-alvo específico (por exemplo, políticos, profissionais de saúde, sociedade civil)
Resumir as evidências em formato curto (por exemplo, uma página) pode facilitar a absorção dos resultados com mensagens principais destacadas para o processo decisório
Utilizar recursos visuais como infográficos
Considerar meios de disseminação direcionados como podcasts, YouTube, LinkedIn, Twitter, ResearchGate e comunicados à mídia
Atualização
Atualizar periodicamente a síntese
Apresentar logo no início da síntese atualizada “O que alterou para esta versão” (por exemplo, data e estratégia de busca, resultados)

Fonte: Adaptado de World Health Organization (WHO)^5^.

**Tabela 2:** Produção de sínteses de evidências realizadas durante a pandemia de covid-19 por tipo de estudo.

Títulos das sínteses elaboradas	n (%)
Busca prévia na literatura	2 (2)
Evidências científicas sobre a eficácia e a segurança do *booster* de doses de vacinas para covid-19	
Nova terapia anticorpo monoclonal para o tratamento da covid-19	
Inventário de referências	21 (23)
Recomendações internacionais de necrópsia em caso de óbitos por covid-19	
Associação entre uso de inibidores da enzima conversora de angiotensina (IECA), enzima conversora de angiotensina 2 (IECA2) e Ibuprofeno e segurança de pacientes infectados por covid-19	
Efeitos das medidas de distanciamento social durante a pandemia da covid-19	
Alternativas terapêuticas farmacológicas para casos leves de covid-19	
Eficácia da utilização de veículos aéreos não tripulados na desinfecção de locais públicos	
Efeito do tratamento profilático farmacológico para covid-19	
Efeito do tratamento profilático farmacológico para covid-19: atualização	
Efeitos do tratamento farmacológico para fase inicial da covid-19 (até cinco dias do início dos sintomas)	
Efeitos da cloroquina e hidroxicloroquina para tratamento pré-hospitalar e/ou ambulatorial de pacientes com covid-19	
Efeitos da ivermectina para tratamento pré-hospitalar ou ambulatorial de pacientes com covid -19	
Efeitos da nitazoxanida para tratamento pré-covid-19	
História natural da covid-19	
Eficácia, efetividade e segurança do tratamento farmacológico de pacientes com quadro leve e moderado de covid-19	
Revisões sistemáticas sobre fatores de agravamento e mortalidade por covid-19	
Segurança sanitária em eventos esportivos no contexto da pandemia da covid -19	
Avaliação da eficácia e segurança da vacinação contra covid-19 em gestantes e puérperas	
Eficácia e a segurança da quarta dose da vacina contra covid-19: 1ª atualização	
Eficácia e a segurança da quarta dose da vacina contra covid-19: 2ª atualização	
Ocorrência de miocardite em crianças após vacinação contra covid-19	
Temas de pesquisa priorizados para covid-19 para subsidiar o lançamento da chamada pública de vacinas	
Eficácia e segurança da vacina coronavac	
Nota técnica	10 (11)
Cloroquina ou hidroxicloroquina (associadas ou não à azitromicina) para o tratamento precoce de pacientes com covid-19 e suas 13 atualizações	
Ocorrência de manifestações clínicas pós-covid-19	
Evidências e recomendações internacionais quanto à vacinação contra covid-19 em crianças com idade entre 5 e 11 anos	
Ocorrência de manifestações clínicas pós-covid-19: atualização	
O uso do exame de tempo de atividade de protrombina na covid-19	
Efeitos do medicamento ciclesonida como auxílio no enfrentamento do covid-19	
Efeitos do uso de gargarejo com batidas de iodo para covid-19 e esclarecimento viral precoce entre pacientes com covid-19 quando gargarejam com povidona-iodo e óleos essenciais	
vDossiê técnico-científico sobre o uso do dióxido de cloro para covid-19	
Estudo de viabilidade da adoção do protocolo de tratamento integral ambulatorial contra a covid-19	
Uso de *corynebacterium parvum* no contexto da covid-19	
Revisão sistemática rápida	31 (34)
Alternativas terapêuticas para tratamento do coronavírus humano	
Alternativas terapêuticas para tratamento do coronavírus humano:1ª atualização	
Alternativas terapêuticas para tratamento do coronavírus humano: 2ª atualização	
Eficácia e segurança de cloroquina e hidroxicloroquina (isoladas ou em associação) para tratamento de infecções por coronavírus humano	
Eficácia e segurança de cloroquina e hidroxicloroquina (isoladas ou em associação) para tratamento de infecções por coronavírus humano: 1ª atualização	
Eficácia e segurança de cloroquina e hidroxicloroquina (isoladas ou em associação) para tratamento de infecções por coronavírus humano: 2ª atualização	
Eficácia e segurança de cloroquina e hidroxicloroquina (isoladas ou em associação) para tratamento de infecções por coronavírus humano: 3ª atualização	
Eficácia e segurança de cloroquina e hidroxicloroquina (isoladas ou em associação) para tratamento de infecções por coronavírus humano: 4ª atualização	
Eficácia e segurança de cloroquina e hidroxicloroquina (isoladas ou em associação) para tratamento de infecções por coronavírus humano: 5ª atualização	
Efetividade de distanciamento social em epidemias	
Eficácia e segurança de plasma convalescente para tratamento de infecções por SARS-CoV-2	
Eficácia da desinfecção de máscaras/respiradores faciais n95, PFF2 e PFF3 para reuso seguro na prevenção de infecções respiratórias	
Eficácia e segurança de plasma convalescente para tratamento de infecções por SARS-CoV-2: 1ª atualização	
Atividade viral de coronavírus humanos em superfícies domésticas e hospitalares	
Eficácia e segurança de plasma convalescente para tratamento de infecções por SARS-CoV-2: 2ª atualização	
Efeitos sanitários e não sanitários das medidas de distanciamento social durante a pandemia da covid-19	
Eficácia e segurança de plasma convalescente para tratamento de infecções por SARS-CoV-2: 3ª atualização	
Efeitos do tratamento farmacológico para fase inicial da covid-19 (até 5 dias do início dos sintomas)	
Efeito do tratamento profilático farmacológico para covid-19	
Eficácia e segurança de plasma convalescente para tratamento de infecções por SARS-CoV-2: 4ª atualização	
Revisão sistemática rápida sobre efeitos da cloroquina e hidroxicloroquina para tratamento pré-hospitalar e/ou ambulatorial de pacientes com covid-19	
Revisão sistemática rápida sobre casos de reinfecção pelo SARS-CoV-2	
Revisão sistemática rápida sobre resposta imunológica ao SARS-CoV-2	
Revisão sistemática rápida sobre casos de reinfecção pelo SARS-CoV-2: 1ª atualização	
Eficácia, efetividade e segurança do tratamento farmacológico de pacientes com quadro leve e moderado de covid-19	
Identificação de fatores de risco para agravamento e mortalidade por covid-19	
Avaliação da eficácia e segurança da vacinação contra covid-19 em gestantes e puérperas	
Segurança da vacinação em adolescentes	
Efetividade e durabilidade da resposta imunológica contra SARS-CoV-2	
Testagem seriada para covid-19 em ambiente escolar	
Eficácia e segurança da vacina coronavac em crianças	
Revisão sistemática tradicional	1 (1)
Eficácia/efetividade do uso de máscaras faciais médicas e não-médicas, em ambientes abertos ou fechados, para imunizados (vacinados e/ou convalescentes) para covid-19	
Sínteses rápida para políticas	1 (1)
Síntese rápida para políticas sobre estratégias para retorno gradual do distanciamento social	
Sumário de resumos	22 (25)
Vitamina D e infecções por SARS-CoV-2	
Limiar de temperatura para triagem em ambientes de convívio	
Distância física mínima recomendada em ambientes de convívio	
Mapeamento de definições de distanciamento social no contexto da pandemia de covid-19	
Mapeamento de definições de caso de covid-19	
Orientações para isolamento no contexto da covid-19	
Mapeamento de termos para definir gravidade de casos de covid-19	
Período alvo para testagem de assintomáticos contactantes de casos confirmados de covid-19	
Mapeamento de evidências sobre alterações fisiopatológicas no transporte aéreo de pacientes com covid-19 e outras doenças respiratórias	
Mapeamento de evidências sobre recomendações nacionais e internacionais de vacinação de gestantes, puérperas, lactantes e crianças para covid-19	
Eficácia do uso de máscaras faciais para prevenção da covid-19	
Efeitos da vacinação de gestantes, puérperas, lactantes e crianças para covid-19	
Mapeamento de evidências sobre recomendações nacionais e internacionais de vacinação de gestantes, puérperas, lactantes e crianças para covid-19	
Identificar o intervalo ideal de testagem para diferenciar eventos de síndrome gripal por covid-19	
Mapeamento de definições de síndrome pós-covid-19	
Eficácia da campanha de vacinação de covid-19 para redução de ocupação de leitos em UTI	
Eficácia e segurança da vacinação contra covid-19 em adolescentes	
Eficácia e segurança da vacinação contra covid-19 em adolescentes: 1ª atualização	
Estratégias nacionais e internacionais de certificação de imunização contra covid-19	
Efetividade da campanha de vacinação de covid-19 para redução de ocupação de leitos em unidades de terapia intensiva	
Identificar e sumarizar estudos que avaliem a eficácia e segurança comparativa de tratamentos para covid-19	
Complementação referente as ações técnicas efetivas no estudo da vitamina D para o combate à covid-19	
Alternativas não farmacológicas à covid-19	

UTI: unidade de terapia intensiva.

Para torná-lo utilizável, fez-se necessário um melhor empacotamento do conhecimento. Além do formato tradicional, uma versão adicional era elaborada por uma equipe de comunicação científica e design de informação, responsável por tornar a linguagem e formato adequados e acessíveis ao público-alvo. Reformulou-se jargões científicos em linguagem simples e investiu-se em resumos visuais, como infográficos (Figura 2).

**Figura 2:** Exemplo de infográfico gerado dos resultados de um inventário de referências. Nota: o inventário de referência tinha o objetivo de identificar e categorizar artigos publicados e estudos em andamento sobre os efeitos da profilaxia farmacológica para a covid-19. Esse estudo não realiza análise de resultados principais, tampouco avalia a qualidade metodológica dos artigos incluídos. Ainda, não representa uma recomendação oficial do Ministério da Saúde sobre a temática em questão.

Utilizou-se constantemente um intermediário do conhecimento para transferência das evidências geradas, no intuito de promover uma maior absorção do conhecimento pelos potenciais usuários^16^. Era um comunicador neutro – como um canal necessário – entre o gerador e o potencial usuário do conhecimento. Pessoas que operam como intermediários podem preparar o caminho para a absorção e potencial implementação do conhecimento científico na política, devido à sua credibilidade^20^.

Um exemplo de situação em que a síntese de evidência apontou ser contrária ao uso de um medicamento e não foi seguida por alguns gestores brasileiros foi o caso da hidroxicloroquina no tratamento da covid-19^24^. Em contraste, um exemplo de implementação das sínteses elaboradas foi o sumário de resumos sobre mapeamento de definições de síndrome pós-covid-19, que contribuiu para adoção da terminologia relacionada a condições pós-covid e definição de novo CID (Classificação Estatística Internacional de Doenças e Problemas Relacionados com a Saúde)^25^.

Um informe diário de evidências científicas sobre tratamento farmacológico para covid-19 foi estabelecido como forma de disseminação. Os estudos eram triados, sumarizados e classificados por delineamento metodológico, bem como avaliados em sua qualidade metodológica e risco de viés, por meio de ferramentas adequadas para cada tipo de estudo, e, por fim, disponibilizados em um painel (Figura 3). Dessa forma, resumos de publicações técnico-científicas de revistas indexadas e em pré-impressão sobre eficácia, segurança e efetividade de medicamentos e produtos biológicos usados para tratamento e prevenção da covid-19 foram disponibilizados diariamente. No início da pandemia, quando havia a ebulição de publicações e divulgações pela mídia, o intuito dessa iniciativa foi promover acesso a evidências de forma qualificada, facilitada e fidedigna.

**Figura 3:** Painel de evidências sobre tratamento farmacológico e vacinas para covid-19.

Havia recursos organizacionais suficientes para responder às solicitações dos tomadores de decisão. Apesar de parte da equipe utilizar computadores e conexão de internet pessoais, devido à adoção do trabalho remoto, havia capacidade instalada no departamento para armazenamento de dados e acesso às fontes de informação, mediante pagamento de licença de uso para as publicações em revistas indexadas.

O grupo buscou reduzir a duplicação de esforços na condução das sínteses, por meio de buscas por iniciativas de pesquisa em andamento em todo o mundo e que abordassem as dúvidas dos tomadores de decisão brasileiros. Por exemplo, o grupo acessou a Rede de Evidências covid-19 (covid-end) para apoiar a tomada de decisões e a Rede Brasileira de Políticas Informadas por Evidências (Evipnet-Brasil). Também era elaborado um protocolo prévio e, quando possível, registrado em plataformas públicas.

### Desafios e Perspectivas na Elaboração de Sínteses de Evidências

Usar síntese de evidências auxilia o processo decisório, mas não é suficiente para garantir a tomada de decisão informada por evidências. Interesses políticos e econômicos interferem no processo, logo, a presença de lideranças institucionais que valorizem o uso de evidências facilita a sua adoção^27^. Além disso, presenciava-se um cenário de saberes incipientes e, consequentemente, presença de incertezas.

Experimentou-se uma espécie de “explosão de evidências” e muitas organizações começaram a se autopromover com a produção de documentos baseados nelas. Não havia consenso metodológico com relação à síntese e avaliação dessas evidências, e os tomadores de decisão nem sempre eram capazes de diferenciar uma revisão sistemática de uma seleção manual de estudos.

Foi comum que diversos grupos de trabalho no país desenvolvessem os mesmos temas. Faz-se necessário melhorar a articulação e as ferramentas de comunicação nacional, bem como haver uma mobilização para priorização de temas e disseminação dos resultados, evitando duplicidade de esforços na elaboração das sínteses de evidências.

Foi solicitado que a equipe lidasse com diferentes fontes de informações, uma vez que havia formuladores de políticas interessados em identificar evidências relacionadas a documentos institucionais de diferentes países, para compreender como foi sua experiência no manejo da pandemia.

A tradução de uma síntese de evidências ou documentos elaborados em outros idiomas para o português foi um recurso pouco usado ou demorado. Também havia falta de uma plataforma on-line disponível para produzir documentos vivos, e tão logo os esforços de pesquisa tornavam-se obsoletos.

Pesquisadores habituados com a produção científica entendem como as evidências evoluem, de modo que suas alterações são vistas como parte do processo. Para o público leigo, esse processo pode ser confuso e causar desconfiança. Assim, uma questão enfrentada foi a ausência de estratégias para lidar com os estudos que relatam resultados do início da pandemia, que poderiam não ser mais relevantes, dados os conhecimentos sobre a situação mais recente da covid-19. Por exemplo, mantinha-se uma revisão rápida sobre o distanciamento social na transmissão do novo coronavírus e os estudos continuavam a ser publicados, relatando os resultados do início da pandemia. No entanto, como se sabia muito mais sobre a transmissão do vírus, bem como sobre as medidas de prevenção e infecção, esses estudos poderiam não fornecer valor relevante.

Um dos grandes desafios do departamento foi engajar atores sociais interessados nos temas das sínteses de evidências, especialmente devido aos prazos limitados. Tal envolvimento poderia ajudar a identificar necessidades de saúde, problemas de pesquisa, formar avaliações e interpretar as descobertas. Por exemplo, eles podem fornecer sua perspectiva sobre a questão, os resultados, a interpretação da análise e o resumo em linguagem simples. A maioria tem interesse em várias áreas de pesquisa, mas nem todos possuem experiência com síntese de evidências. Uma estratégia útil seria fornecer, para os interessados, treinamento adequado sobre conceitos e termos de saúde baseada em evidências^28^.

Destaca-se que diante dos recursos disponíveis, a equipe sempre buscou tomar as decisões mais assertivas para o cenário, mas outros esforços também precisam ser incentivados. Além de contribuições para elaboração de sínteses, é necessário informar a população sobre os resultados das sínteses e reconhecer uma evidência científica confiável. Iniciativas nessa área podem contribuir para democratização da ciência e aprimoramento do pensamento crítico sobre informações divulgadas na internet.

Além disso, embora as evidências sejam fundamentais para informar decisões, não são o único fator considerado na tomada de decisão. Contudo, acredita-se que a falta de alinhamento entre as esferas de governo pode ter comprometido a implementação das sínteses.

## CONSIDERAÇÕES FINAIS

Um passo importante para a aplicação do conhecimento científico em tempo real no Brasil foi dado durante a pandemia da covid-19. Não basta investir em pesquisas selecionadas, é preciso também incentivar o uso e a criação de plataformas online vivas. Para isso, é primordial que se trabalhe em rede para evitar a sobreposição de esforços e a não disseminação do conhecimento produzido. Mais pesquisas são necessárias sobre como apresentar e priorizar os resultados e como engajar as partes interessadas em sínteses rápidas, para garantir que todas as perspectivas sejam alcançadas. Assim, destaca-se a necessidade de institucionalização do uso transparente e sistemático de evidências em processos decisórios. Esse caminho percorrido durante a pandemia pela equipe pode ser considerado uma proposta de modelo inovador no que tange ao uso de sínteses de evidências para apoio à tomada de decisões em saúde.
